# Supplementary material for: The polyHIS Tract of Yeast AMPK Coordinates Carbon Metabolism with Iron Availability
Source: Int J Mol Sci. 2023 Jan 10;24(2):1368. doi: 10.3390/ijms24021368 (PMC9863760; doi:10.3390/ijms24021368)
Supplement: Supplementary file 1 [file ijms-24-01368-s001.zip › NEW SUPPLEMENTARY/Supplemental Figure Legends.docx]

**Supplementary Figure Legends**

**Figure S1 related to figure 1. A**. Schematic diagram showing domains and regions of Snf1, with important residues and motifs. Aa1-53 is referred to as the pre-kinase region (**PKR**) containing a polyHIS tract at Aa18-32 which functions as a pH sensing module (**PSM**). Aa54-391 comprises the kinase domain (**KD**) – mutation of K84R prevents ATP-binding (kinase dead) and the activation loop phosphorylation site is at T210. Together the PKR and KD comprise the N-terminal domain (**NTD**). The regulatory domain (**RD**) comprises aa392-633 and comprises the entire C-terminal domain (**CTD**); The γ-subunit Snf4 binds to the auto-inhibitory sequence (AIS) (**RD-γ**) to prevent it from inhibiting the kinase domain – mutation of L470 inactivates the auto-inhibitory sequence. The β-subunits Sip1, Sip2 or Gal83 bind the far C-terminus (**RD-β**). **B.** Rate of *ADH2* expression in Snf1-GFP and Snf1^ΔH^-GFP cells in ethanol media treated with 1mM EDTA for 3 hours. N=3. Error bars are ±1 standard deviation. T-tests were performed as indicated. NS = not significant, * = p≤0.05, ** = p≤0.01, *** = p≤0.001. **C, D**. Quantification of Western blot data for Snf1 abundance (**C)** and phosphorylation at T210 (**D**) under indicated conditions. Data from **Figures 1C, 6A** and **6E** was used. Error bars are ±1 standard deviation. T-tests were performed as indicated. NS = not significant, * = p≤0.05, ** = p≤0.01, *** = p≤0.001.

**Figure S2 related to figure 2. A.** Protein alignment of Aft1 and Aft2. Amino acids 16 and 24 (inclusive) that are deleted in Aft1^Δ9^ are underlined in green. Amino acids 16 to 36 (inclusive) that are deleted in Aft1^Δ24^ are enclosed in a dark red box. A basic region unique to Aft1 is indicated by a purple box. A polyQ region unique to Aft1 is indicated by a blue box. **B**. Rate of *prFET3*::LacZ expression upon addition of 100μM BPS for 3 hours in *aft1Δ* cells in glucose media with indicated *AFT1* plasmids or empty vector. N=3. Error bars are ±1 standard deviation. T-tests were performed as indicated. NS = not significant, * = p≤0.05, ** = p≤0.01, *** = p≤0.001. **C**. Rate of *prFET3*::LacZ expression in wild-type cells upon addition of 100μM BPS for 3 hours in 4% glucose versus 3% glycerol media. N=3. Error bars are ±1 standard deviation. T-tests were performed as indicated. NS = not significant, * = p≤0.05, ** = p≤0.01, *** = p≤0.001.

**Figure S3 related to figure 4. A.** Rate of *prFET3*::LacZ expression of *aft1Δ* cells expressing Aft1, Aft1^AA^ (S210A, S224A) or Aft1^DD^ (S210D, S224D) upon addition of 100μM BPS for 3 hours. N=3. Error bars are ±1 standard deviation. T-tests were performed as indicated. NS = not significant, * = p≤0.05, ** = p≤0.01, *** = p≤0.001. **B**. Cells from **Figure 4C** were allowed to grow for an additional 24 hours. Media was **not** refreshed. **C**. Quantification of **Figure 4C**. **D.** Quantification of **Figure S3B**. Error bars are ±1 standard deviation. T-tests were performed as indicated. NS = not significant, * = p≤0.05, ** = p≤0.01, *** = p≤0.001.

**Figure S4 related to figure 6**. **A.** Quantification of **Figure 6F.** Error bars are ±1 standard deviation. T-tests were performed as indicated. NS = not significant, * = p≤0.05, ** = p≤0.01, *** = p≤0.001.
