# Supplementary figures and images for: The polyHIS Tract of Yeast AMPK Coordinates Carbon Metabolism with Iron Availability
Source: Int J Mol Sci. 2023 Jan 10;24(2):1368. doi: 10.3390/ijms24021368 (PMC9863760; doi:10.3390/ijms24021368)

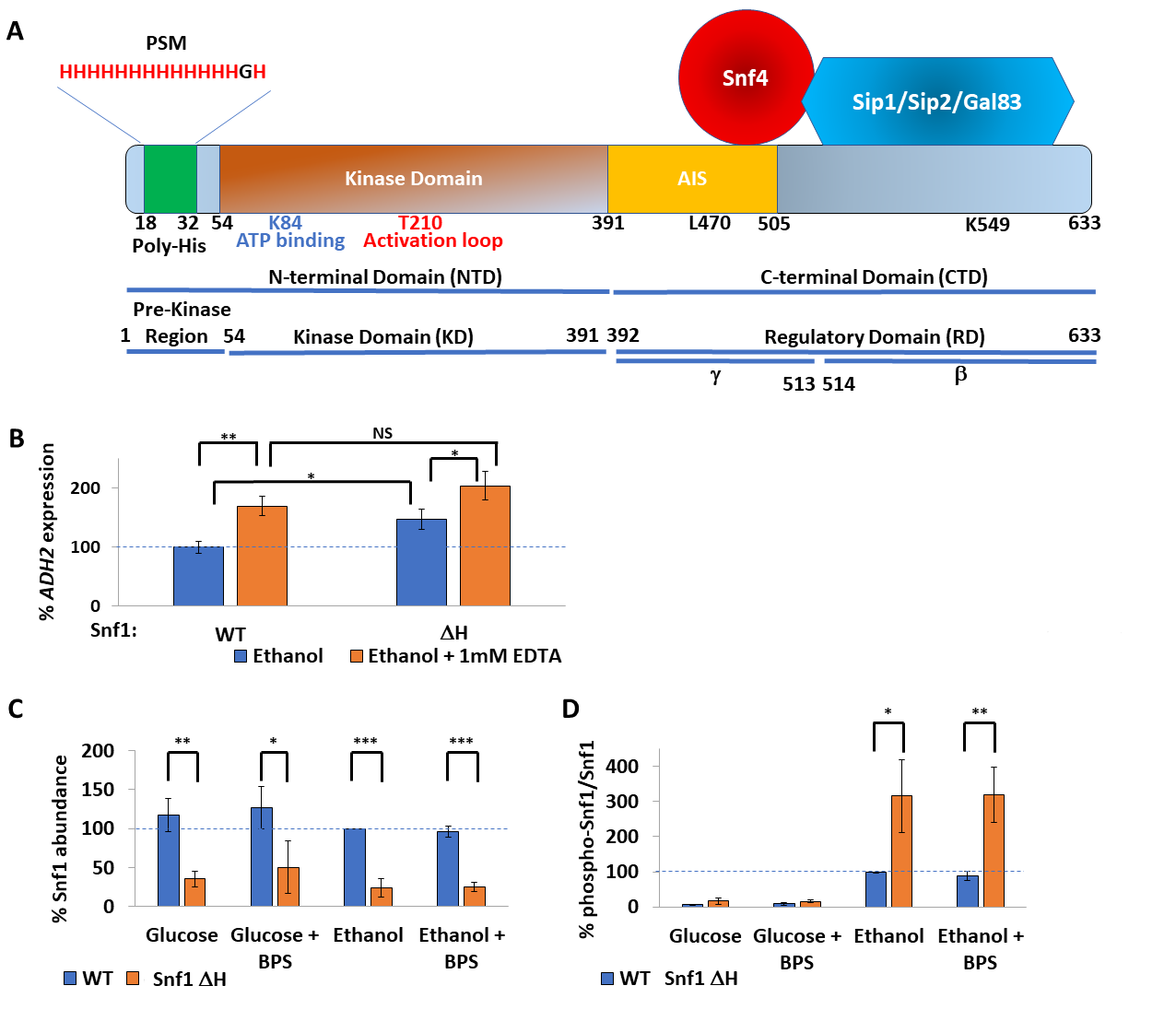

Supplement: Supplementary file 1 [file ijms-24-01368-s001.zip › NEW SUPPLEMENTARY/Figure S1.tif]

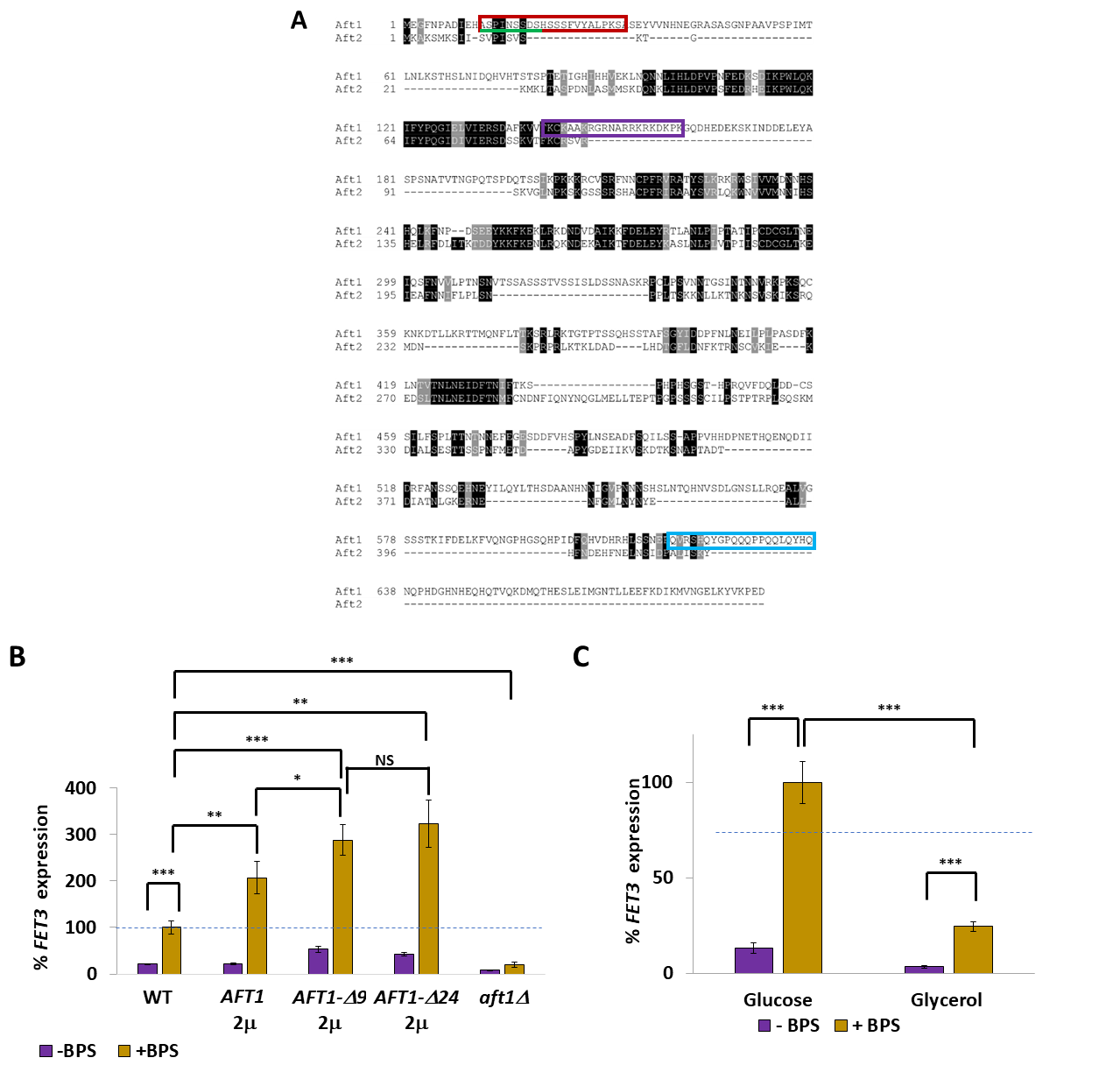

Supplement: Supplementary file 1 [file ijms-24-01368-s001.zip › NEW SUPPLEMENTARY/Figure S2.tif]

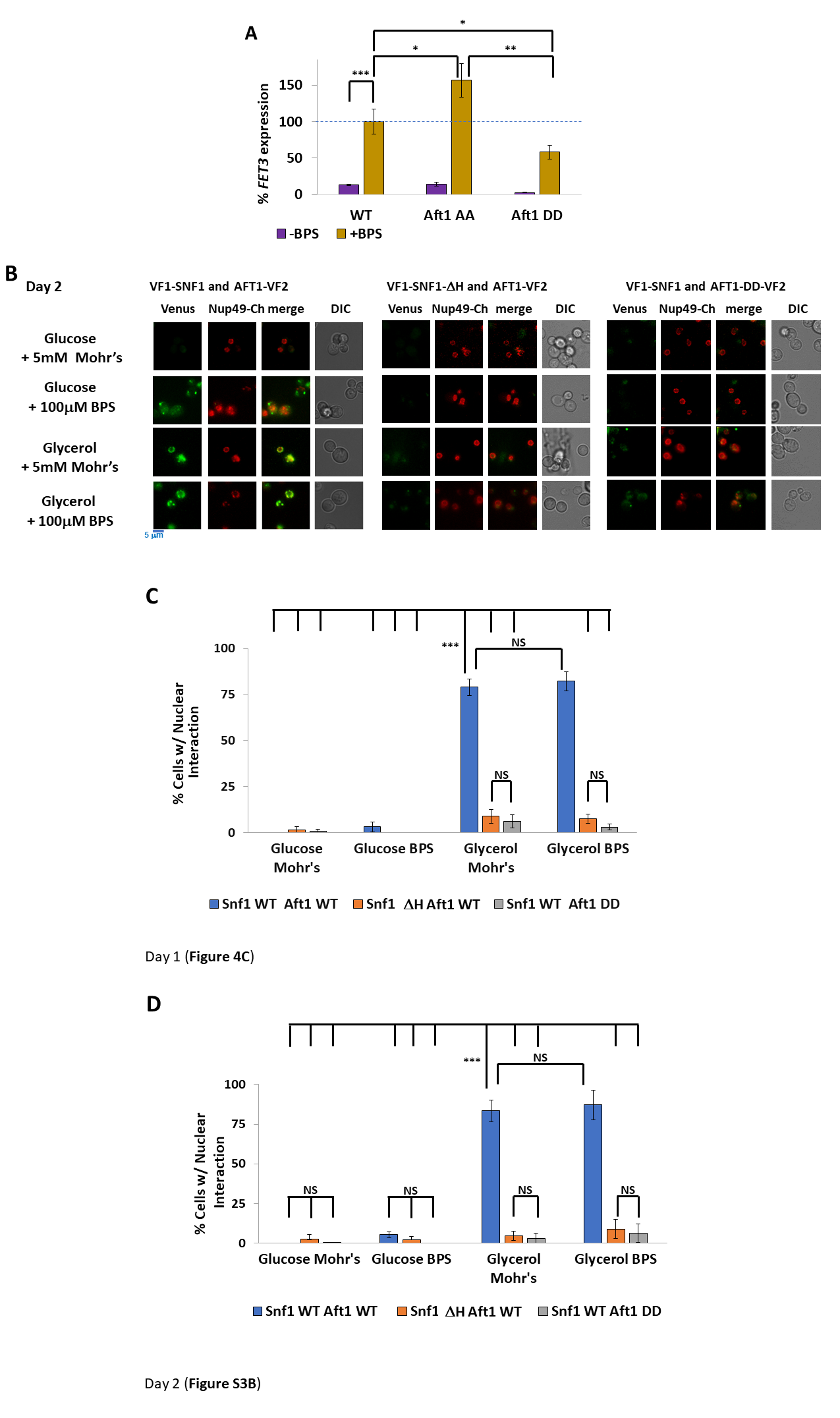

Supplement: Supplementary file 1 [file ijms-24-01368-s001.zip › NEW SUPPLEMENTARY/Figure S3.tif]

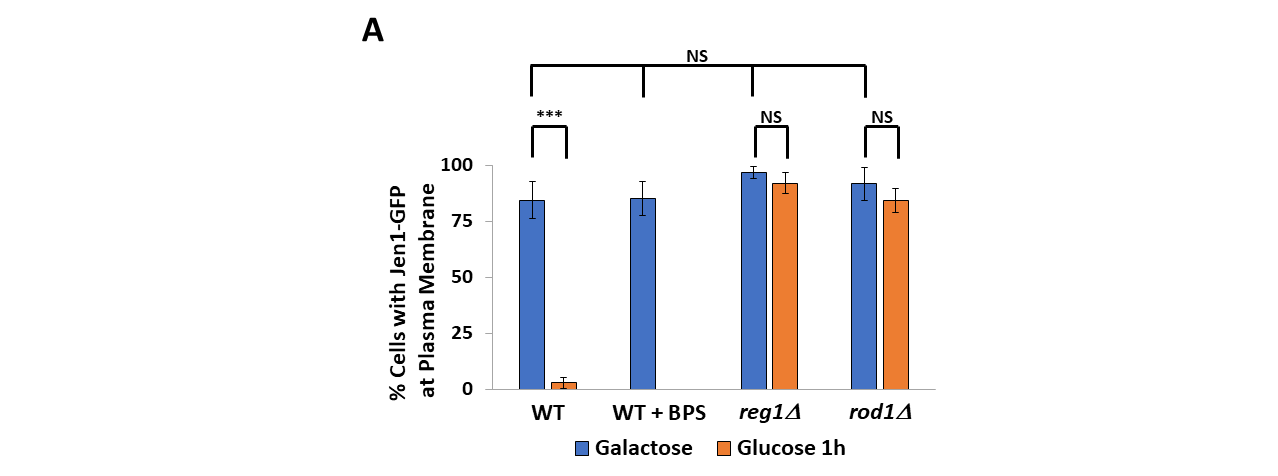

Supplement: Supplementary file 1 [file ijms-24-01368-s001.zip › NEW SUPPLEMENTARY/Figure S4.tif]
